# Invited Editorial: Waiting Room Care is Not the Solution to Emergency Department Boarding

**DOI:** 10.1016/j.acepjo.2025.100161

**Published:** 2025-05-05

**Authors:** Andrew J. Bouland, Juan A. March

**Affiliations:** Division of Emergency Medical Services, Department of Emergency Medicine, Brody School of Medicine, East Carolina University, Greenville, North Carolina, USA

**Keywords:** patient safety, emergency medicine, waiting room, return visits

In this issue, an article by Gaudet et al^1^ entitled “Patients Evaluated and Discharged from the Waiting Room Do Not Have a Higher Rate of 72-Hour Returns - A Retrospective Observational Study” evaluated return rates of patients in the emergency department (ED). They compared patients who were assessed and treated only in the ED waiting room vs patients placed directly in a treatment area vs patients initially placed in the waiting room and then moved to a treatment area.^1^ The authors should be commended for investigating this topic as the concept of “waiting room medicine” emerges as a new practice setting for emergency physicians nationwide. There exists limited literature on the topic of waiting room medicine, with existing studies examining the effects of crowding rather than patients who were assessed and treated only in the waiting room.^2^, ^3^, ^4^, ^5^

This study found no difference in the rate of 72-hour return between patients treated in the waiting room only compared with traditional treatment spaces, suggesting that ED triage is effective at selecting patients for waiting room-only evaluation. Furthermore, it suggests that emergency physicians can correctly identify and assess patients in the waiting room to determine which patients can be safely discharged. However, it is important to note that this process may vary by institution, and in departments with nurse-only triage, it is dependent on the experience and skill of the individual triage nurse.

The practice of waiting room medicine is an emerging concept in emergency medicine and warrants additional study. Emergency physicians nationwide have been versatile and adaptive to this new practice, as demonstrated by our ability to safely assess and treat patients in any setting, including a chair in the waiting room. Yet, prior research has shown that evaluation in a chair or hallway limits the physician’s assessment, delays care, and prolongs the length of stay (LOS).^6^^,^^7^ For instance, abdominal examinations are limited when the patient is sitting upright,^8^ and genitourinary examinations may be deferred due to a lack of privacy. Certain sensitive patient histories (ie, sexual history and HIV status) may also be skipped in waiting room assessments due to a lack of privacy. Additionally, as mentioned in this article, this study did not evaluate patient or clinician experience, which may be adversely affected by evaluations in the waiting room where there is often limited space, comfort, and privacy.^9^, ^10^, ^11^

A major limitation of this study is its single-center design, especially considering its urban setting and exclusion of suburban and rural sites. The study also missed patients who may have been pronounced dead at the scene by Emergency Medical Services after ED discharge and thus not transported back to the ED, or patients who may have been treated and released by Emergency Medical Services after their initial ED visit. In contrast to this study, one previous large retrospective nationwide study found that patients placed in hallways had higher odds of elopement, 72-hour ED revisits, and 10-day ED revisits compared with patients placed in regular ED rooms.^12^ The results of this study must also be interpreted with caution, as the patients who were evaluated in the waiting room only had lower Emergency Severity Index scores. These results cannot be extrapolated to suggest that higher acuity patients can be appropriately assessed and treated in the waiting room.

Unfortunately, this study does not answer a major question regarding patients treated primarily in the waiting room and then admitted to an inpatient service. Do the patients who are admitted to an inpatient service following assessment and treatment in the “waiting room-only” have increased LOS, morbidity, and 30-day mortality compared with treatment in other locations? For patients treated in the waiting room who require admission, are emergency physicians missing diagnoses or interventions that affect the patient further in their clinical course? These questions must be topics of further investigation.

Many clinical scenarios question whether waiting room-only treatment is the solution for patients who are ultimately admitted. Are septic patients treated solely in the waiting room receiving a prompt diagnosis and timely administration of intravenous antibiotics and a crystalloid bolus? Are we delaying the diagnosis and treatment of appendicitis, diverticulitis, and gastrointestinal bleeds because of the inability to perform a proper physical examination in a chair? Are we missing important physical examination findings in genitourinary examinations?

Although this study attempts to analyze waiting room medicine, it has not proven that it is safe for our patients. In addition, this study does not address the root cause of ED boarding, which results from delays in transferring admitted ED patients to inpatient beds. Unfortunately, prolonged ED boarding results in extended waiting room times for higher acuity ED patients, resulting in treatment delays, suboptimal patient care, and patients dying in our waiting rooms before they can be evaluated.^13^, ^14^, ^15^, ^16^ We can only hope that the development of a new measure on the tracking of ED boarding by the Centers for Medicare and Medicaid Services, with input from the American College of Emergency Physicians, will eventually result in improved patient outcomes, ED operations, and health care costs.^17^

## Funding and Support

By *JACEP Open* policy, all authors are required to disclose any and all commercial, financial, and other relationships in any way related to the subject of this article as per ICMJE conflict of interest guidelines (see www.icmje.org). The authors have stated that no such relationships exist.
